# The University of California San Francisco (UCSF) Training Program in Implementation Science: Program Experiences and Outcomes

**DOI:** 10.3389/fpubh.2020.00094

**Published:** 2020-03-27

**Authors:** Priya B. Shete, Ralph Gonzales, Sara Ackerman, Adithya Cattamanchi, Margaret A. Handley

**Affiliations:** ^1^Division of Pulmonary and Critical Care Medicine, Zuckerberg San Francisco General Hospital and Trauma Center, University of California, San Francisco, San Francisco, CA, United States; ^2^Department of Medicine, University of California, San Francisco, San Francisco, CA, United States; ^3^Department of Social and Behavioral Sciences, University of California, San Francisco, San Francisco, CA, United States; ^4^Center for Vulnerable Populations at Zuckerberg San Francisco General Hospital and Trauma Center, University of California, San Francisco, San Francisco, CA, United States; ^5^Department of Epidemiology and Biostatistics, University of California, San Francisco, San Francisco, CA, United States; ^6^Division of General Internal Medicine at Zuckerberg San Francisco General Hospital and Trauma Center, Department of Medicine, University of California, San Francisco, San Francisco, CA, United States

**Keywords:** implementation science training, research education, implementation science competencies, curriculum evaluation, on-line education

## Abstract

**Purpose:** We evaluated outcomes of trainees who have completed the Certificate program in Implementation Science at the University of California San Francisco.

**Methods:** All students who completed the in-person Certificate Program between 2008 and 2015 (*n* = 71), or the online Certificate Program between 2016 and 2017 (*n* = 13), were eligible for our study. We assessed the potential impact of the Certificate Program on the professional development of trainees, through participant surveys on their self-reported level of comfort with pre-defined competencies, and on academic productivity.

**Results:** Of eligible trainees, 54 in-person (77%) and 13 online (100%) Certificate Program participants completed surveys. In-person trainees reported a total of 147 implementation science-related publications in peer-reviewed journals (median 3 publications/trainee, IQR 1–15). Thirty-four trainees (63%) reported being a Principal Investigator (PI) of 64 funded implementation science-related grants (median 2 grants/trainee, IQR 1–4). Fifteen percent (15%, *n* = 8) of participants reported receiving an NIH grant on which they were the PI, including R01 or P01 level funding (*n* = 4, 7%) and K awards (*n* = 3, 6%). Both in-person and online trainees reported median high to moderate confidence for all 12 competencies assessed. Confidence waned in skills aligning with later stages of implementation research for all trainees.

**Conclusion:** The moderate to high confidence in all competencies assessed and reported high level of academic productivity support the benefits of intensive, graduate-level training focused on applied methods to support career development of implementation scientists.

## Contributions to the literature

- Methodological approach to evaluating implementation science training programs.
- Analysis and data interpretation that demonstrates the feasibility of an implementation science training program in achieving core competencies as measured by productivity and self-reported proficiency.
- Demonstration of the feasibility of online and in-person platforms for delivering high quality implementation science training that integrates didactics with longitudinal mentorship and project-based learning.

## Introduction

Despite the growing body of research and evidence-based guidelines, there is still a significant lag in translating research into practice. Implementation science (ImS), the study of the methods used to design, implement, and evaluate strategies to increase uptake of evidence-based interventions, has never been more relevant (1). Researchers and policymakers around the world increasingly recognize the need for frameworks, theories, or models to design and evaluate interventions that address programmatic performance gaps in context-specific ways (2). Research funding agencies are now more often requiring frameworks, theories or models in grant proposals, and non-academic stakeholders involved in process planning and evidence translation are also using these tools (3). For these reasons, it is essential to ensure that physicians, researchers, public health practitioners, and others working to translate evidence into practice are trained in implementation science.

The field of implementation science has had its own “performance gaps” related to the availability of resources and training programs to acquire relevant knowledge and skills (4). To address these gaps, several types of programs have been developed that offer training in a variety of formats: degree granting programs, stand-alone certificate programs, short course mentored immersion programs, and workshops. Apart from our own University of California San Francisco (UCSF) Implementation Science Training Program (5), several other programs are affiliated and administered through academic institutions (6–8). Of these, several offer a multi-year, non-degree conferring fellowship experience to students that often includes longitudinal mentorship (6), while others use a shorter 1, 2 day workshop format as an introduction to the implementation science as a field of study (7). Additional training programs, such as the National Institutes of Health Training Institute for Dissemination and Implementation Research in Health (TIDIRH) (9, 10), have been developed and administered through national institutes, often in collaboration with multiple academic centers. These institutes have resulted from national prioritization of support for dissemination and implementation research and increase in funding mechanisms for implementation science proposals. TIDIRH and similar training programs are centered around core-competencies that reflect training priorities in implementation science (11, 12). The majority of implementation science training programs are offered in a short-course format (e.g., summer institute or 1, 2 week intensive course) and integrate a “practical” or project-based experience linked to close mentorship as part of their core curricula (13). While some of the shorter course programs are described as having led to additional training modules and potentially degree conferring curricula, the progress and results have not yet been published in the literature (7). While we focused here on programs with descriptions or outcomes published in the peer-reviewed literature, a more comprehensive list of training programs is available on the Society for Implementation Research Collaboration website (14).

**Figure 1:**
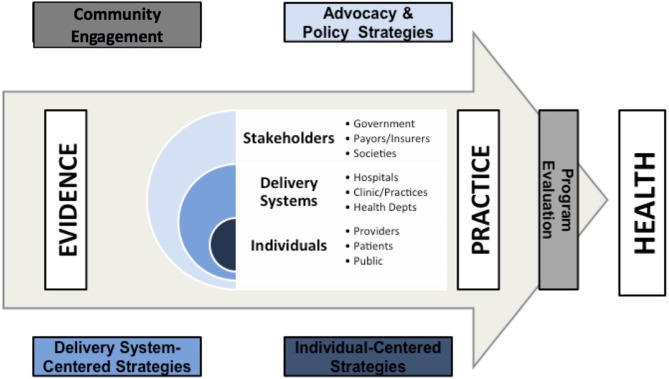
An ecological overview of the process of practice improvement.

We developed the UCSF Implementation Science Certificate program in 2008 to provide trainees with knowledge, skills and experience in applying implementation science methods. We have focused on developing a curriculum around competencies in implementation science processes rather than specific frameworks or theories. Frameworks are later introduced as tools to help facilitate the process of community engagement, intervention design, intervention evaluation, and policy translation (Figure 1). We have previously described the conceptual framework of this training program, which focused on three core principles: (1) behavior change among individuals and health care systems is fundamental to effectively translating evidence into practice; (2) engaging with a wide range of individuals and stakeholder organizations is essential for achieving effective and sustainable change; and (3) implementation and dissemination must be iterative and dynamic (5). These principles highlight that evidence is consumed by inter-related groups of stakeholders, delivery systems, and individuals.

The six, 10 week (equivalent to an academic quarter) long courses in the program focus on how to align priorities among and between players in these three domains and how to address barriers within each domain in order to successfully translate evidence into practice and ultimately into improved health (Figure 1). Table 1 describes the six courses mapped to the key training domains and competencies that were included in the design of the program. The original training program was delivered in an in-person format either as a track within the UCSF Master's in Clinical Research (TICR) program or as a stand-alone Implementation Science Certificate Program. In 2016, we introduced an online format for the 6 courses to make both the courses and Certificate Program available to a wider audience within and outside the United States. Both the in-person and online courses (Appendix Table) are centered around didactics to convey key course content. In addition, each course uses learner-selected, project-based assignments to facilitate the learner's skill development and build personal research projects through the assignments, and the program provides longitudinal faculty-level mentorship for trainees enrolled in implementation science courses no matter their overall track. Implementation Science Certificate courses are credit- bearing and require tuition, paid through either a larger program of study such as the Masters in Clinical Research, through tuition for other graduate programs at UCSF, through participant grants, or through out of pocket payment. Our Certificate program provides a very “high touch” approach to implementation science training in which learners receive feedback from course faculty on a weekly basis on the application of concepts taught to their own projects. Most courses are taught by lead faculty in collaboration with 2–6 additional faculty as co-Instructors or small group facilitators. Each course anchors assignments to a longitudinal project that each learner come up with independently. This allows learners to apply newly-learned methods and skills to improve and develop their own projects.

**Table 1:** Revised domains and competencies for implementation sciences (ImS) training programs, with examples of relevant activities and courses offered by the training in clinical research program (TICR) at the University of California, San Francisco, 2011–2012.

**Domain**	**Competency**	**Relevant activities**	**Relevant courses**
Team Science	1. Develop a collaborative, multidisciplinary team that shares a common language, and promotes a transdisciplinary blending of disciplines. 2. Engage in collaborative writing, including the production of grants and manuscripts that meet the unique needs of sponsors of implementation and dissemination sciences.	- Small-group, multidisciplinary works in progress seminars - Grant applications and manuscript submissions - Mock study sections	Introduction to Implementation Science Theory and Design Designing Interventions to Change Organizational Behavior Community-Engaged Research Designing Individual-Level Implementation Strategies Translating Evidence into Policy Grant Writing Course (seminar)
Context Identification	3. Determine the range of factors—behavioral, social, ethical, institutional, political, economic, historical—that inform the research question, and design structure	- Small-group, multidisciplinary works-in-progress seminars - Field internship program - Multidisciplinary faculty mentorship	Community-Engaged Research Designing Individual-Level Implementation Strategies Designing Interventions to Change Organizational Behavior
Literature identification and assessment	4. Identify relevant theory, evidence, methods, • and perspectives outside the clinical domain of the research program.	- Literature search and retrieval skills	All Clinical Research courses (TICR) available teach literature search skills All Implementation Science Courses
Community engagement	5. Build relationships with community members • and community-based organizations, in order to engage multiple perspectives on the problem	- Field internship program - Individual research and implementation projects	Community-Engaged Research Translating Evidence into Policy
Intervention design and research implementation	6. Integrate diverse disciplinary, stakeholder and community perspectives into a cogent intervention design and/or implementation and dissemination strategy. 7. Utilize a comprehensive implementation framework to guide the integration of theory with specific intervention, evaluation, and dissemination activities	- Small-group, multidisciplinary works-in-progress seminars. - Biostatistical consultations	Introduction to Implementation Science Theory and Design Designing Individual-Level Implementation Strategies Designing Interventions to Change Organizational Behavior
Evaluation of effect of translational activity	8. Employ epidemiological methods in study designs, program evaluations and causal inference. 9. Gain facility with qualitative and quasi- experimental designs to plan, implement, and evaluate interventions and policy impact. 10. Determine and measure processes and outcomes that support iterative cycles of implementation and bidirectional flow of information.	- Epidemiology courses - Biostatistics courses (including quasi-experimental designs)	Translating Evidence into Policy Program Evaluation in Clinical and Public Health Settings
Behavioral change communication strategies	11. Disseminate research/program results to relevant stakeholders and communities in a manner that maximizes their influence and sustainability outside of the research paradigm 12. Ability to articulate Implementation Science as an innovative approach to clinical and community-based research.	- Field internship program	- Translating Evidence into Policy - Designing Individual-Level Implementation Strategies

Here, we sought to assess feasibility of our mutli-faceted implementation science training program by reviewing outcomes of trainees who have completed (1) the in-person UCSF Master's in Clinical Research Implementation Science Track; (2) the UCSF in-person Implementation Science Certificate Program; or (3) the UCSF online Implementation Science Certificate Program as of 2017. We focus on productivity in implementation science-related activities for the in-person Masters track and Certificate Program participants, and comfort level with implementation science-related competencies for all participants.

## Methods

### Participants

Eligible participants included all students who completed 4 or more courses in the in-person Masters in Clinical Research Implementation Science Track or Implementation Science Certificate Program between 2008 and 2015, or the online Implementation Science Certificate Program in 2016–2017. An introductory course and a course on community engagement were required by all participants in the certificate program, while other certificate requirements could be filled by any of the courses available. Individuals who took a la carte courses but completed fewer than the 4 required to receive a certificate were excluded.

### Procedures

In order to assess the potential impact of the training program on professional development, we conducted a cross-sectional study that surveyed participants on their self-reported competency with core components of implementation science training and on academic productivity.

### Competency Questionnaire

We sent online surveys assessing comfort with implementation science competencies across 12 domains (see Additional Files) to all participants who completed either the Certificate Program regardless of mode of instruction (on-line or in-person). The competencies assessed were those identified using a Delphi process as key skills required for implementation science practitioners when developing our training program (5).

### Academic Productivity

Additionally, we sent online surveys assessing demographics and academic productivity to all participants. Program faculty contacted non-respondents once via email to encourage survey completion. Questions regarding academic productivity survey was limited to in-person students who completed the in-person Certificate Program because the online program only began in 2016–2017 (participants would not have had time to submit manuscripts or grant proposals and receive notice of outcome between completion of the training in mid 2017 and the time of the surveys in late 2017).

### Measures

Competency was assessed using a questionnaire (see Online Supplement) that requested participants to rate their confidence in 12 implementation science-related competencies that represented the goals of the UCSF Implementation Science Training Program (5), with responses following a Likert scale ranging from 1 (No Confidence) to 5 (Total Confidence). The questionnaire also had space for participants to clarify reasons behind their self-reported competency ratings. Included competencies were: developing a multi-disciplinary team for ImS-related research; collaborative writing for grants and publications; identifying multi-level influences across social, behavioral, economic, and historical spheres relevant to research topics; understanding and applying theories, evidence, and methods outside of the clinical research domain; building relationships with community members to engage in diverse perspectives on the research topic; integrate diverse perspectives into cogent intervention designs and/or implementation strategies; utilizing a comprehensive implementation framework to guide the integration of theory into interventions; applying epidemiological methods in design, evaluation, and causal inference; gaining facility in use of qualitative and quasi-experimental designs; selecting, and measuring process outcomes that can support an iterative approach to intervention design and evaluation; disseminating results in collaboration with relevant stakeholders outside of traditional research dissemination strategies; articulating implementation and dissemination sciences approaches as distinct from clinical research.

The demographic and academic productivity survey (see Additional Files) collected information on participant demographics (gender and race/ethnicity) and current job title and institutional affiliation. In addition, the survey asked in-person course participants to provide information on funded grants (as Principal Investigator or Co-Investigator), implementation-science related publications in peer-reviewed journals or publications in implementation science-related peer-review journals, and/or awards or honors received since completion of the Implementation Science Masters Track or Certificate Program.

### Data Analysis

We described trainee characteristics, competency ratings, and productivity measures using either proportions and 95% confidence intervals (CI) or medians with inter-quartile ranges (IQR). Differences in median competency rating between in-person and online trainees were not evaluated due to small sample size of the online cohort. Qualitative data including optional comments provided by participants in the questionnaire were categorized according to competency. All surveys were created and disseminated using Survey Monkey (Survey Monkey, San Mateo CA). Data analysis was performed using Stata version 14 (Stata Corporation, College Station, TX), and Excel (Microsoft Corp, Redmond, WA).

## Results

### Survey Participation

Of 71 eligible trainees who completed the in-person Certificate Program, 54 (77%) completed both the demographic and academic productivity survey and the post-training competency survey. Participation was similar for in-person trainees who completed the Master's in Clinical Research Implementation Science Track and the stand-alone Implementation Science Certificate Program (26/32, 81% vs. 28/39, 72%). All 13 eligible online Implementation Science Certificate Program trainees completed the demographic portion of the demographic and productivity survey and the post-training competency survey.

### Demographic Characteristics

The majority (74%) of in-person trainees were female, 46% were non-Hispanic white and 40% were Asian (Table 2). Only 5 (9%) in-person trainees were from groups underrepresented in medicine, including 2 black or African American and 3 Hispanic trainees. The majority of in-person trainees (67%) reported their current position as academic faculty at the Assistant Professor or Associate Professor level and, as expected for an in-person program, most (78%) were currently based at UCSF. The characteristics of the online program trainees reflect a different demographic makeup of participants, with 84% being female, and 54% who identified as black or African American. Although the online trainee survey did not capture the specific job or academic title of participants, it did show that 6 (46%) had faculty appointments at the time of their ImS training, 4 (31%) were still trainees, and 2 (15%) were staff. Five (38%) of the online trainees were from outside of the US, and 5 of the 8 (63%) online trainees from the US were not based at UCSF (Table 2).

**Table 2:** Trainee demographics, current positions and institutions.

**Characteristic**	**In-person trainees** **(*N* = 54)** *N* (%)^*^	**On-line trainees** **(*N* = 13)** *N* (%)^*^
**Gender**		
Females	40 (74)	11 (85)
Males	14 (26)	2 (15)
**Ethnicity**		
Hispanic or Latino	3 (6)	1 (8)
Not Hispanic or Latino	51 (94)	12 (92)
**Race**		
American Indian or Alaskan Native	0	0
Asian	22 (41)	2 (15)
Black or African American	2 (4)	6 (46)
White	28 (52)	4 (31)
More than one race	2 (4)	0
Declined to answer	0 (0)	1 (8)
**Education at time of ImS training**		
MD	44 (82)	5 (38)
PhD	5 (9)	6 (46)
MPH, other Master's, or Bachelor's only	5 (9)	7 (54)
**Current position**		
Medical student	1 (2)	–
Post-doctoral fellow	7 (12)	–
Assistant professor	25 (4)	–
Associate professor	11 (2)	–
Professor	3 (5.5)	–
Clinician (non-academic)	3 (5.5)	–
Staff, lecturer or other	4 (7)	–
**Institution of primary appointment**		
UCSF	35 (65)	3 (23)
UCSF-affiliated delivery system	7 (13)	0
Other delivery system	3 (6)	0
US Universities other than UCSF	6 (11)	5 (38)
International	2 (3)	5 (38)
Other	1 (1)	0

* *Percent may not equal 100% due to rounding*.

### Productivity in Implementation Science for In-person Trainees

Trainees reported a total of 181 publications which included implementation science methods or focus areas (median 3 publications/trainee, IQR 1-15) (Table 3). Thirty-four trainees reported being a Principal Investigator (PI) of 64 implementation science-related grants (median 2 grants/trainee, IQR 1–4). Eight (15%) participants reported receiving an NIH grant on which they were the PI. These included R01 or P01 level funding (*n* = 4, 7%) and K awards (*n* = 3, 6%). Nine (17%) of participants were a PI of other federal grants including those administered by the Patient Centered Outcomes Research Institute (PCORI), Health Resources and Services Administration (HRSA), Veteran's Administration (VA), United States Agency for International Development (USAID), and Agency for Healthcare Research and Quality (AHRQ). Of the 34 participants who were a PI on implementation science-related grants, 19 (56%) reported successfully competing for 2 or more awards. Additionally, 20 trainees reported being co-investigators on 38 implementation science-related grants (median 1 grant/trainee, IQR 1–4) (Table 3).

**Table 3:** Academic productivity of in-person trainees (*N* = 54).

**Implementation science-related publications in peer reviewed journals**	***N* (%)**
Total number of publications reported = 181	
None	20 (37)
1–5	26 (48)
6–10	4 (7)
11–15	3 (6)
16–40	0
>40	1 (2)
**Implementation science-related grant funding**	
Principal investigator	
Any ImS-related grant^a^	34 (63)
NIH ImS-related grant	8 (15)
NIH individual K award	3 (6)
NIH R03	2 (4)
NIH R21	2 (4)
NIH R01 or P01	4 (7)
Other federal ImS-related grant	9 (17)
PCORI grant	3 (6)
HRSA, VA, USAID, or AHRQ	6 (11)
UCSF intramural ImS-related grant	14 (26)
Foundation or other ImS-related grant	16 (30)
Co-investigator^b^	
Any ImS-related grant	26 (48)
NIH ImS-related grant	20 (37)
NIH R21	2 (4)
NIH R01 or PO1	6 (11)
Other federal ImS-related grant	
PCORI grant	3 (6)
HRSA,VA, USAID, or AHRQ	4 (7)
UCSF intramural ImS-related grant	4 (7)
Foundation or professional society	4 (7)

a *Nineteen respondents reported receipt of ≥2 grants as Principal Investigators across all mechanisms*.

b *Respondents could report receipt of multiple grants across all mechanisms*.

### Self-Reported Confidence With Implementation Science-Related Skills for In-person Trainees

The median level of competence was reported at 4 (high confidence) for 9 of the 12 competencies assessed, and at 3 or 3.5 (moderate confidence) for the remaining three (Table 4). No trainees reported having “no confidence” in any of the 12 competencies. Seven (13.5%) trainees reported having “low confidence” in Epidemiologic Methods (Competency 8). Five (9.6%) trainees reported having low confidence in the competencies for Qualitative and Quasi-experimental Designs (Competency 9) and Process and Outcome Measurement (Competency 10). No trainees reported having low confidence in any other competency.

**Table 4:** Reported level of confidence in implementation science related competencies^*^.

**Competency**	**In-person trainees** **(*N* = 52)**	**Online trainees** **(*N* = 13)**
	**Median (IQR)**	**Median (IQR)**
1. Develop a collaborative, multidisciplinary team that shares a common language, and promotes a transdisciplinary blending of disciplines.	4 (3, 4)	4 (4, 4)
2. Engage in collaborative writing, including the production of grants and manuscripts that meet the unique needs of sponsors of implementation and dissemination sciences.	4 (3, 4)	4 (3, 4)
3. Determine the range of factors behavioral, social, ethical, institutional, political, economic, historical that inform the research question, and design structure.	4 (3, 4)	4 (3, 4)
4. Identify relevant theory, evidence, methods, and perspectives outside the clinical domain of the research program.	3 (3, 4)	4 (3, 4)
5. Build relationships with community members and community-based organizations, in order to engage multiple perspectives on the problem.	4 (3, 4)	4 (3, 5)
6. Integrate diverse disciplinary, stakeholder and community perspectives into a cogent intervention design and/or implementation and dissemination strategy.	4 (3, 4)	3 (3, 4)
7. Utilize a comprehensive implementation framework to guide the integration of theory.	4 (3, 4)	3 (3, 4)
8. Employ epidemiological methods in study designs, program evaluations, and causal inference.	3.5 (3, 4)	3 (3, 4)
9. Gain facility with qualitative and quasi-experimental designs to plan, implement, and evaluate interventions and policy impact.	4 (3, 4)	3 (3, 4)
10. Determine and measure processes and outcomes that support iterative cycles of implementation and bidirectional flow of information.	3 (3, 4)	3 (3, 4)
11. Disseminate research/program results to relevant stakeholders and communities in a manner that maximizes their influence and sustainability outside of the research paradigm.	4 (3, 4)	3 (3, 4)
12. Articulate ImS as an innovative approach to clinical and community-based research.	4 (3, 4)	3 (3, 4)

*[Competencies ranked from no confidence (1) to total confidence (5)] ImS- Implementation Science*.

* *Medians based on data for 52 of 54 respondents for in-person trainees and 13/13 respondents for online trainees. Two in-person respondents did not complete this portion of the survey*.

### Self-Reported Confidence With Implementation Science-Related Skills for Online Trainees

The median level of proficiency following certificate training was reported at 4 (high confidence) for 5 of the 12 competencies assessed, and at 3 or 3.5 (moderate confidence) for the remaining 7 (Table 4). Six trainees (46%) reported having “low confidence” in at least one competency. The number of participants who rated level of comfort as low for these competences ranged between 1 and 3, with the same individuals rating their confidence as “low” in multiple competencies: Determine Contextual Factors for Research (Competency 3) (*n* = 1, 8%); Theory and Perspectives (Competency 4) (*n* = 2, 15%); Implementation Frameworks (Competency 7) (*n* = 2, 15%); Epidemiologic Methods (Competency 8) (*n* = 2, 15%); Qualitative and Quasi-experimental Designs (Competency 9) (*n* = 2, 15%); Process and Outcome Measurement (Competency 10) (*n* = 1, 8%); Dissemination (Competency 11) (*n* = 2, 15%); Articulate ImS Approaches to Others (Competency 12) (*n* = 3, 23%).

Qualitative data from open comments in these surveys pointed to a trend toward lower confidence in some competencies due to not yet having the opportunity to apply the knowledge and skills acquired in projects. Representative comments to the open-ended question seeking additional comments about the trainee's confidence in implementation science-related competencies include:

“*I feel that I have a lot of great knowledge learned from this certificate program, but now I need application practice in real life to increase my confidence in my abilities.”*

“*I have had an opportunity to gain understanding of the implementation science concepts and apply them in my day to day work and thought as I plan for my current and future research career and interests. [I] am currently providing guidance and mentorship in my region where the understanding of implementation science research is still very low.”*

“*I'm sure they will continue to grow, to the extent that my future work involves opportunities to apply them!”*

## Discussion

This assessment of the UCSF Implementation Science Training program demonstrates the feasibility and success of our approach of providing in-depth training along core implementation science-related competencies for biomedical and public health researchers. Trainees who completed the in-person training program report a high level of productivity and moderate to high confidence in all of the competencies assessed. While this may be related to the general culture of academic rigor and productivity at UCSF, where many in-person trainees were based, the specific nature of the productivity in terms of implementation science related papers and grants suggests an association with our training program. It is worth noting that these competencies, although developed originally in 2012 (5), share almost all core components with other educational competencies subsequently developed for dissemination and implementation research (11). These include competencies that reflect the skills and knowledge necessary for implementation-focused research, with some components being applicable to general clinical and translational research as well. Although productivity data are not yet available, a majority of trainees who completed the online training program also reported moderate to high confidence in all of the competencies assessed. These data confirm that in-depth training delivered either in-person or online can enable trainees from a wide range of backgrounds to apply core implementation science methods and skills.

There have been few evaluations of academic implementation science training programs that report productivity and skills-based competency assessments. The National Institute of Mental Health (NIMH) and Veteran's Administration (VA) co-funded Implementation Research Institute (IRI) training program has also reported high level of participant satisfaction with their curriculum. IRI is an interdisciplinary training program that provides didactic training, faculty mentoring (both local and distance), support and guidance for pilot research and grant writing through 2 2 week on-site training sessions followed by site visits and long-distance mentorship. Fellows in the program are established PhD or MD investigators in mental health with an interest in implementation science. The 31 participants in 3 cohorts surveyed have received 52 funded awards in implementation science and authored 208 publications (15). While these data may reflect the already advanced nature of this cohort, a subsequent study using social network analysis done 5 years later by IRI showed that continued longitudinal mentorship established by the training program resulted in long-term impact in scientific productivity, including significantly more grant submissions, and publications (13). Results from an evaluation of the Mentored Training for Dissemination and Implementation Research in Cancer (MT-DIRC), based out of Washington University St. Louis, showed significantly improved implementation science skills as reported by trainees at 6 and 18 months post-training aligned to 43 competencies (16). MT-DIRC trains fellows and experienced investigators (junior to mid-level faculty) who are pivoting toward implementation science methods through an intensive 5 day course paired with longitudinal mentorship. Significant training is also available to improve mentorship for mentors in the program. The results of our evaluation of the UCSF Implementation Science Training Program parallel the results of these programs, especially those linked to high-quality longitudinal mentorship which is a hallmark of our program as well. Chambers and colleagues point out in their mapping of dissemination and implementation training needs, the effect of longitudinal mentorship on successful training depends in part of the experience of the learner, the goals of the training program, and the infrastructure to support research locally (17). While mentorship seems to be a unifying characteristics of successful training programs, one challenge in the field has been comparing programs that have been evaluated based on differing outcomes; not all evaluation studies report on the same or even similar productivity and competency outcomes those developed and used in our training program. Our program links mentorship, didactics and project-based learning to competencies, providing directed learning over longer periods of time.

The productivity of our graduates in a short amount of time is a testament to the overall effectiveness of our program in training implementation science researchers. However, our evaluation revealed some areas for improvement that other training programs may also be able to learn from. Competencies for which the majority of trainees reported low or low-moderate confidence were related to later stages of implementation research. For example, the competency related to Qualitative and Quasi-experimental Designs (Competency 9) and Process and Outcome Measurement (Competency 10) fall within the training domain, “Evaluation of effect of translational activity.” We suspect this result is in part because most trainees were at an early stage of their career and were still in the process of developing interventions rather than conducting intervention evaluations. Nonetheless, these data point to the need for further training in study designs and measures for conducting evaluation of implementation interventions in real-world settings. In addition, self-rated competency was generally lower for online trainees. Potential reasons include that less time had passed, on average, between completion of the training and the competency assessment and that a higher proportion of online trainees were at an earlier stage of their career. Nonetheless, we are considering various methods to increase live contact between course faculty and online trainees to provide additional support (18). We have also developed courses that focus on these topics which launched this in Spring 2019.

Our results suggest that a hybrid model that combines didactics with focused, project based assignments and longitudinal mentorship is a successful model for implementation science training. Our results demonstrate the feasibility of both in-person and online training platforms for research training. Both training platforms effectively enhanced competency of learners in implementation science skills. This finding parallels other comparisons of online vs. in-person trainings (19, 20), in which the competency and skills delivered over the short- to medium term are approximately the same between modes of delivery. The longitudinal distance mentorship in our training program likely offset typical critiques of lack of personal interaction within online platforms. To adapt our in-person to an online program, didactics were video-recorded and available on our learning platform. All in-person content is available to online students, and assignments, were identical. We cultivated participant interaction through online small group sessions (“sync” sessions), moderated by faculty discussants who work with the same group of students through the semester. Finally, both in-person and online participants benefited from the availability of real-time discussion boards to facilitate knowledge sharing. These characteristics are particularly well-suited for implementation science training for early career investigators and seasoned academics who are pivoting their research portfolios (17).

In addition, as demonstrated by the diverse demographic of our online cohort as compared to our in-person cohorts, online training has a broader reach. Over 50% of our on-line trainees identified as Black African or African American compared to only 4% in our in-person program. This reflects high levels of enrolment from researchers in sub-Saharan African and students within our Research in Implementation Science for Equity program, which is a sponsored ImS training program for underrepresented in medicine (URM) junior faculty from all over to complete ImS training.

There are a few limitations of our program evaluation thus far. First, the sample size of participants surveyed is small overall, particularly for online trainees, making comparisons between online, and in-person cohorts difficult to interpret. Our online program is the newest addition to our training platform and, as should be a part of any continued program evaluation, we plan on further assessment of competencies and productivity as additional online trainees complete the Certificate Program. Second, a more in-depth qualitative assessment could have enabled identification of additional strengths and weaknesses, including reasons for low ratings by some trainees for some of the competencies. Further research is needed to determine whether short, intensive training programs alone, with or without follow-up longitudinal components, can achieve similar results, and whether there is significant variation in productivity and competency that arise as a result of different instructional platforms among different trainee populations. Finally, it is possible that our results on productivity or competency for in-person trainees may be skewed because of the non-response rate, if all non-responders were less productive with grant applications or publications. However, we had over 75% response rate, making such a bias unlikely.

In summary, this evaluation of our training program has validated the feasibility of our approach to competency-based implementation science training. Although it requires a significant time commitment from both faculty and trainees, the results of our “high-touch” program in terms of self-rated competence and academic productivity suggest that our approach is successful at developing implementation science researchers. Our program has the added benefit of mentorship aligned along core competencies through each course that focuses on project-based learning as a way to enhance sustainable scholarly productivity in implementation science.

## Conclusions

Academic implementation science training through an intensive, graduate-level program can generate competent, productive implementation science researchers. A longitudinal training program using applied methods to support career development of implementation scientists can be delivered effectively in both an in-person and online learning format.

## Data Availability Statement

The datasets generated and/or analyzed in this study are not publicly available due to the personal nature of demographic and identifying information from course participants surveyed as part of a course evaluation, but are available from the corresponding author on reasonable request.

## Ethics Statement

The studies involving human participants were reviewed and approved by The UCSF Committee on Human Research a (IRB ##16-20323). Written informed consent for participation was not required for this study in accordance with the national legislation and the institutional requirements.

## Author Contributions

PS and MH analyzed and interpreted the survey data regarding trainee demographics, productivity, and competencies. PS wrote the manuscript with major contributions from MH and AC. RG defined core training competencies. SA provided significant contribution in the design of surveys and in training program curriculum development. All authors read and approved the final manuscript.

### Conflict of Interest

The authors declare that the research was conducted in the absence of any commercial or financial relationships that could be construed as a potential conflict of interest.

## Supplementary Material

Additional File 1 UCSF Implementation Science Training Program Academic Productivity Survey. This survey was administered to in-person Certificate Program participants to gauge their productivity in academic implementation science after completion of their training.

Additional File 2 UCSF Implementation Science Training Program Skills and Competency Survey. This survey was administered to all Certificate Program participants to characterize their self-reported competency with skills and methods in implementation science after completion of their training.

Additional File 3 STROBE Checklist.

Appendix Table Courses included in UCSF Implementation Science Certificate Training Program.

## Abbreviations

AHRQ: Agency for Healthcare Research and Quality
CI: Confidence Interval
HRSA: Health Resources and Services Administration
ImS: Implementation Science
IQR: Interquartile Range
NIH: National Institutes of Health
NIMH: National Institute of Mental Health
PCORI: Patient Centered Outcomes Research Institute
PI: Principal Investigator
TICR: Training in Clinical Research
TIDIRH: Training Institute for Dissemination and Implementation Research in Health
UCSF: University of California San Francisco
USAID: United States Agency for International Development
VA: Veteran's Administration.
