# Supplementary material for: The University of California San Francisco (UCSF) Training Program in Implementation Science: Program Experiences and Outcomes
Source: Front Public Health. 2020 Mar 27;8:94. doi: 10.3389/fpubh.2020.00094 (PMC7118197; doi:10.3389/fpubh.2020.00094)
Supplement: Additional File 1 — UCSF Implementation Science Training Program Academic Productivity Survey. This survey was administered to in-person Certificate Program participants to gauge their productivity in academic implementation science after completion of their training. [file Data_Sheet_2.PDF]

ImS

Tracking of Alumni/Students:
UCSF Implementation Science (ImS) Training Program

Survey Goal

Dear Students/Alumni,
We would like to track your career progress to help support funding/renewal applications for the UCSF Implementation Science (ImS) Training Program. Please complete the brief survey below by Oct. 15. We appreciate your time. If you would prefer not to receive the survey in future years, please let us know.

Margaret Handley and Adithya Cattamanchi
UCSF ImS Training Program co-Directors

Tracking of Alumni/Students:
UCSF Implementation Science (ImS) Training Program

* 1. What is your name?

* 2. Which of these NIH-defined* racial categories below best describes you? Please choose only one.
* http://grants.nih.gov/grants/funding/women_min/race_ethnicity_qa.htm#3744

	Females	Males	Sex/Gender Not Reported
Hispanic or Latino	○	○	○
Not Hispanic or Latino	○	○	○
NA	○	○	○

* 3. Which of these NIH-defined* ethnic categories below best describes you? Please choose only one.
* http://grants.nih.gov/grants/funding/women_min/race_ethnicity_qa.htm#3744

	Females	Males	Sex/Gender Not Reported
American Indian/Native Alaskan	○	○	○
Asian	○	○	○
Native Hawaiian or Other Pacific Islander	○	○	○
Black or African American	○	○	○
White	○	○	○
More Than One Race	○	○	○
Unknown or NA	○	○	○

* 4. Current Position (Choose All Appropriate Answers)

- [ ] Student
- [ ] Resident
- [ ] Fellow
- [ ] Research Associate
- [ ] Instructor
- [ ] Lecturer
- [ ] Assistant Professor
- [ ] Associate Professor
- [ ] Professor
- [ ] Clinician
- [ ] Other (please specify)

* 5. Current institution (Primary Appointment)

- [ ] UCSF
- [ ] SFGH
- [ ] SFVA
- [ ] CHORI
- [ ] Other (please specify)

* 6. Have you received any funded grants as PI or co-PI since you have finished your ImS training?

- ( ) Yes
- ( ) No

7. If so, what type and how many ImS-related grants have you received as PI or co-PI?

	1	2	3	4	5	6	7	8	9	10
UCSF internal grant	☐	☐	☐	☐	☐	☐	☐	☐	☐	☐
NIH K12	☐	☐	☐	☐	☐	☐	☐	☐	☐	☐
NIH K23	☐	☐	☐	☐	☐	☐	☐	☐	☐	☐
NIH R03	☐	☐	☐	☐	☐	☐	☐	☐	☐	☐
NIH R21	☐	☐	☐	☐	☐	☐	☐	☐	☐	☐
NIH R01 or P01	☐	☐	☐	☐	☐	☐	☐	☐	☐	☐

Other (please specify type and number of funded grants)

* 8. Have you received any funded ImS related grants as Co-Investigator since you finished your ImS training?

- Yes
- No

9. If so, how many and what type of funded ImS grants have you received as Co-Investigator?

	1	2	3	4	5	6	7	8	9	10
UCSF internal grant	☐	☐	☐	☐	☐	☐	☐	☐	☐	☐
NIH K12	☐	☐	☐	☐	☐	☐	☐	☐	☐	☐
NIH K23	☐	☐	☐	☐	☐	☐	☐	☐	☐	☐
NIH R03	☐	☐	☐	☐	☐	☐	☐	☐	☐	☐
NIH R21	☐	☐	☐	☐	☐	☐	☐	☐	☐	☐
NIH R01 or P01	☐	☐	☐	☐	☐	☐	☐	☐	☐	☐

Other (please specify type and number of funded grants)

* 10. Number of ImS-related publications in peer-reviewed journals

11. Please list any other ImS-related awards, products, media coverage or other accomplishments.

Thank you for your time and feedback!

ImS
