# Supplementary material for: The University of California San Francisco (UCSF) Training Program in Implementation Science: Program Experiences and Outcomes
Source: Front Public Health. 2020 Mar 27;8:94. doi: 10.3389/fpubh.2020.00094 (PMC7118197; doi:10.3389/fpubh.2020.00094)
Supplement: Additional File 2 — UCSF Implementation Science Training Program Skills and Competency Survey. This survey was administered to all Certificate Program participants to characterize their self-reported competency with skills and methods in implementation science after completion of their training. [file Data_Sheet_3.PDF]

UCSF ImS Competency Assessment

Survey Goal

Dear Students/Alumni,

We would like to track your self-reported competency attainment as it relates to completion of your ImS training. Please complete the brief survey below by Oct 15th. We appreciate your time. If you would prefer not to receive the survey in future years, please let us know.

Margaret Handley and Adithya Cattamanchi
UCSF ImS Training Program co-Directors

ImS

UCSF ImS Competency Assessment

*** 1. What is your name?**

*** 2. The following best describes your ability to:**

	No confidence	Low confidence	Moderate confidence	High Confidence	Total confidence
Develop a collaborative, multidisciplinary team that shares a common language, and promotes a transdisciplinary blending of disciplines.	○	○	○	○	○

*** 3. The following best describes your ability to:**

	No confidence	Low confidence	Moderate confidence	High Confidence	Total confidence
Engage in collaborative writing, including the production of grants and manuscripts that meet the unique needs of sponsors of implementation and dissemination sciences.	○	○	○	○	○

*** 4. The following best describes your ability to:**

	No confidence	Low confidence	Moderate confidence	High Confidence	Total confidence
Determine the range of factors—behavioral, social, ethical, institutional, political, economic, historical — that inform the research question and design structure.	○	○	○	○	○

*** 5. The following best describes your ability to:**

	No confidence	Low confidence	Moderate confidence	High Confidence	Total confidence
Identify relevant theory, evidence, methods, and perspectives outside the clinical domain of the research program.	○	○	○	○	○

*** 6. The following best describes your ability to:**

	No confidence	Low confidence	Moderate confidence	High Confidence	Total confidence
Build relationships with community members and community-based organizations, in order to engage multiple perspectives on the problem.	○	○	○	○	○

*** 7. The following best describes your ability to:**

	No confidence	Low confidence	Moderate confidence	High Confidence	Total confidence
Integrate diverse disciplinary, stakeholder and community perspectives into a cogent intervention design and/or implementation and dissemination strategy.	○	○	○	○	○

*** 8. The following best describes your ability to:**

	No confidence	Low confidence	Moderate confidence	High Confidence	Total confidence
Utilize a comprehensive implementation framework to guide the integration of theory.	○	○	○	○	○

*** 9. The following best describes your ability to:**

	No confidence	Low confidence	Moderate confidence	High Confidence	Total confidence
Employ epidemiological methods in study designs, program evaluations and causal inference.	○	○	○	○	○

*** 10. The following best describes your ability to:**

	No confidence	Low confidence	Moderate confidence	High Confidence	Total confidence
Gain facility with qualitative and quasi-experimental designs to plan, implement, and evaluate interventions and policy impact.	○	○	○	○	○

*** 11. The following best describes your ability to:**

	No confidence	Low confidence	Moderate confidence	High Confidence	Total confidence
Determine and measure processes and outcomes that support iterative cycles of implementation and bidirectional flow of information.	○	○	○	○	○

*** 12. The following best describes your ability to:**

	No confidence	Low confidence	Moderate confidence	High Confidence	Total confidence
Disseminate research/program results to relevant stakeholders and communities in a manner that maximizes their influence and sustainability outside of the research paradigm.	○	○	○	○	○

*** 13. The following best describes your ability to:**

	No confidence	Low confidence	Moderate confidence	High Confidence	Total confidence
Articulate IDS as an innovative approach to clinical and community-based research.	○	○	○	○	○

14. Do you have any comments about your confidence in your ImS-training competencies?

Thank you for your time and feedback!

ImS
