# Supplementary material for: The University of California San Francisco (UCSF) Training Program in Implementation Science: Program Experiences and Outcomes
Source: Front Public Health. 2020 Mar 27;8:94. doi: 10.3389/fpubh.2020.00094 (PMC7118197; doi:10.3389/fpubh.2020.00094)
Supplement: Appendix Table — Courses included in UCSF Implementation Science Certificate Training Program. [file Data_Sheet_4.docx]

**Appendix Table. Courses included in UCSF Implementation Science Certificate Training Program**

| Course | Course Goals |
| --- | --- |
| Introduction to Implementation Science Theory and Design | - -Identify and justify medical evidence that is ready for translation; - -Apply a conceptual framework for translating evidence into practice, policy and public health; - -Apply theory and evidence to the design of more effective implementation strategies; - -Evaluate and analyze implementation strategies using a combination of techniques. |
| Community-Engaged Research | - -Describe different approaches to engaging patients, the public, community-based organizations, and other stakeholders in health research, intervention design and implementation. - -Understand the purpose and historical, social and political context of a range of engagement strategies. - -Critically evaluate the benefits and limitations of different approaches to community collaboration in specific contexts. - -Explain how engagement principles and strategies can influence research design, intervention development, data collection and evaluation activities, and implementation. - -Identify a range of potential community partners. - -Develop and justify a conceptual model and detailed plan for incorporating a community partnership into an existing or planned research or implementation project. |
| Designing Individual-Level Implementation Strategies | - -Describe common behavior change theories used to facilitate uptake of health interventions - -Identify behavioral and contextual determinants that can influence health intervention uptake by individuals using behavior change theories - -Design, adapt, and/or tailor an implementation strategy that targets key behavioral and contextual determinants using an intervention design framework - -Create visual representations (e.g., figures and tables) of the logic, functions, and techniques of an implementation strategy to foster individual behavior change. |
| Designing Interventions to Change Organizational Behavior | - -Understand the nature and scope of organizational change tools for promoting the widespread adoption of new clinical and public health interventions. - -Consider how each tool can be applied to address implementation problems in the student's substantive field of expertise. - -Complete a case study that applies selected tools to the student's area of expertise. - -Identify the parts of any healthcare organization, including its structure, culture, system of power and organizational environment. - -Understand what's unique about the healthcare industry and how this contributes to implementation challenges. - -Analyze how the parts of healthcare organizations come together to produce leverage points for change. - -Map barriers and facilitators of the successful implementation of healthcare policies within organizations. - -Understand how strategic managers adapt healthcare organizations to meet the demands of their organizational environments. - -Analyze the potential for implementation gaps and failures, and how to rig systems for implementation success. - -Develop systems change strategies that take advantage of the spontaneous diffusion of innovation. - -Promote collaborative systems change through “bottom-up” strategies that leverage off of the knowledge of front-line workers. |
| Program Evaluation in Clinical and Public Health Settings | - -Explain the main concepts/terms and key elements used in program evaluation; - -Apply logic models and evaluation frameworks to guide evaluation of their chosen program/implementation strategy; - -Select appropriate process and outcome metrics for evaluating their chosen program/implementation strategy; - -Describe qualitative and quantitative approaches to collecting data on process and outcome metrics; - -Develop effective dissemination strategies for the results of program evaluation. |
| Translating Evidence into Policy | - -Determine what problems in health care are "ripe" for a policy solution. - -Identify policy targets at the local, state, national, and international level. - -Locate, interpret and influence existing legislative and administrative policy. - -Map stakeholders around a policy issue and identify strategies to engage supporters or diffuse opponents. - -Build strategic relationships with stakeholders engaged in a policy issue. - -Develop strategies for disseminating research through the media to influence the policy process. - -Organize effective communication strategies with policymakers to influence decision making. - -Design community based participatory research or recognize how research is used in the policy process. - -Plan and engage in community organizing and grassroots advocacy activities. - -Design a logic model to guide implementation and evaluation of a policy campaign. - -Assess your capacity to sustain your participation in the policy campaign. |
